# Single-Trial Kernel-Based Functional Connectivity for Enhanced Feature Extraction in Motor-Related Tasks

**DOI:** 10.3390/s21082750

**Published:** 2021-04-13

**Authors:** Daniel Guillermo García-Murillo, Andres Alvarez-Meza, German Castellanos-Dominguez

**Affiliations:** Signal Processing and Recognition Group, Universidad Nacional de Colombia, Manizales 170003, Colombia; amalvarezme@unal.edu.co (A.A.-M.); cgcastellanosd@unal.edu.co (G.C.-D.)

**Keywords:** functional connectivity, Gaussian kernel, motor imagery, motor execution

## Abstract

Motor learning is associated with functional brain plasticity, involving specific functional connectivity changes in the neural networks. However, the degree of learning new motor skills varies among individuals, which is mainly due to the between-subject variability in brain structure and function captured by electroencephalographic (EEG) recordings. Here, we propose a kernel-based functional connectivity measure to deal with inter/intra-subject variability in motor-related tasks. To this end, from spatio-temporal-frequency patterns, we extract the functional connectivity between EEG channels through their Gaussian kernel cross-spectral distribution. Further, we optimize the spectral combination weights within a sparse-based $\ell_{2}$-norm feature selection framework matching the motor-related labels that perform the dimensionality reduction of the extracted connectivity features. From the validation results in three databases with motor imagery and motor execution tasks, we conclude that the single-trial Gaussian functional connectivity measure provides very competitive classifier performance values, being less affected by feature extraction parameters, like the sliding time window, and avoiding the use of prior linear spatial filtering. We also provide interpretability for the clustered functional connectivity patterns and hypothesize that the proposed kernel-based metric is promising for evaluating motor skills.

## 1. Introduction

Motor imagery (MI) is understood as an act wherein an individual contemplates motor execution’s mental action without apparent action. MI as a higher cortical function is increasingly postulated as an innovative and valid learning tool that allows simulated solutions for motor tasks in the learning phase [1,2]. Alternatively, Motor Execution (ME) is the actual practice of the movement. MI and ME share common sensorimotor areas, and they both involve planning and executing the same motor plan, but their neural mechanisms have some differences [3,4]. In practice, to indicate that motor learning had taken place and retained after a training phase, motor execution performance is measured [5,6]. Moreover, the brain plasticity that is induced by motor learning can be associated with significant changes in electroencephalographic (EEG) features, particularly in the pre-execution phase [7,8]. However, the degree of learning new motor skills varies among individuals, which is mainly due to the between-subject variability in brain structure and function captured by EEG recordings [9]. Thus, because of the inter-session/subject variability, about 15–30% of users do not gain enough control over Brain–Computer Interfaces (or BCI illiteracy) , seriously limiting the widespread use of MI-BCI [10].

A solid reason for BCI illiteracy is that subjects with poor control performance do not exhibit discriminative task-related changes over the modulation of Sensorimotor Rhythms (SMR) within the resting-state as well as in the interval of MI responses [11]. In particular, BCI faces real-world challenges, which are mostly caused by the low spatial resolution of EEG that, along with the nonstationarity present in the recorded neurophysiological signals, results in a poor signal-to-noise ratio (SNR). To enhance SNR, a regular pipeline of MI/ME processing involves the feature extraction stage using spatial filtering to increase mental states’ discriminability, for which several methods are reported, like Riemannian geometry-based algorithm [12], $\ell_{1}$-norm unsupervised Fukunaga–Koontz transform [13], Spectrum-weighted Tensor Discriminant Analysis [14], Bayesian spatio-spectral filter optimization [15], and common spatial patterns (CSP), which maximize the variance of one class to another [16], among others. Although CSP is the most widely employed algorithm, several factors may hinder the extraction of highly separable features in terms of spatial patterns. Namely, while spatial filters are efficient on clean datasets that were obtained in constrained environments, they need a convenient artifact removing from EEG signals because of distorting artifacts and outliers of real-world acquisition contexts [17], the interval of neural responses is generally chosen heuristically. Additionally, features that are extracted by CSP are dense with patterns repeatedly selected [18], small datasets [19], and unsuitability to analyze task-free (unlabeled) EEG data (like resting-state), due to the sources being identified by CSP representing the summed activity from multiple distinct neural electrodes that, together, allow for differentiation by contrasting two or more labeled MI conditions [20]. Consequently, for solving the MI-BCI inefficiency problem, several efforts are to be conducted to extract new MI-related features to obtain meaningful representations with spatially separated patterns, as suggested in [21].

In general, the brain functional connectivity of motor-related tasks is related to synchronization mechanisms within (or sometimes between) different SMR, mainly emerging in the sensorimotor cortex [22]. Thus, most of the approaches for assessing brain regions’ relationships rely on covariance matrices computed from the input EEG signal. Because of the nonlinearity and nonstationarity inherent to the MI neural activity, the baseline Euclidean distance-based covariance estimation tends to be highly inaccurate. For dealing with inter and intra-individual variability, the covariance matrix is enhanced by using mapping approaches, which account for specific geometric relationships through proper distances, and factorize the multivariate EEG data into its stationary nonstationary components [23]. Still, selecting the most appropriate distance to encompass the brain signal variability remains challenging [24]. Meanwhile, using features that were extracted from the EEG dynamic brain network analysis is feasible for increasing the classification accuracy [25,26,27]. Nonetheless, dealing with EEG variability because of evoked nonstationary responses remains challenging, particularly in MI-BCI inefficient subjects [28,29]. Some single-trial functional connectivity measures have been introduced from EEG signals, i.e., Cross-Correlation Coefficient (CCF) and Phase Lag Value (PLV) [30], to support motor-related classification tasks. However, different researchers point to the fact that single-trial connectivity can only be used to obtain more interpretable results, but not satisfactory discrimination [31,32].

Here, we propose a single-trial kernel-based functional connectivity measure as an EEG-based feature extraction method to deal with inter/intra-subject variability in motor-related tasks. To this end, from spatio-temporal-frequency patterns, we extract the functional connectivity between the EEG channels through a novel kernel-based cross-spectral distribution estimator. Namely, the Gaussian kernel is used to compute the pairwise kernel-based channel dependencies because of its universal approximating ability. Further, we optimize the spectral combination weights within a sparse-based $\ell_{2}$-norm as a feature selection framework matching the motor-related labels that perform a dimensionality reduction of the extracted Gaussian Functional Connectivity features. We also interpret the extracted functional connectivity patterns by clustering them into ensembles of individuals with common behavior. Although our proposal is a data-driven approach, as other well-known motor-related feature extraction methods, i.e., CSP [18], it does not require the full trial set to compute discriminative EEG channel dependencies. The latter can benefit the implementation of real-time brain-machine interfaces. We contrast the proposed Gaussian functional connectivity measure with the baseline CSP method, two commonly used single-trial FC measures (Cross-Correlation Coefficient and Phase Lag Value), and more elaborated feature extraction approaches in the state-of-the-art. The validation results that were accomplished in three EEG databases (two with MI and one with ME) show that the proposed connectivity measure allows for reaching very competitive classifier performance with less affected values by feature extraction parameters, like the tuning of sliding time window. Besides, the introduced connectivity measure does not demand a prior linear spatial filtering as a preprocessing procedure. Additionally, the interpretation of subject clusters using our functional connectivity patterns benefits in understanding the inter/intra-subject variability in motor-related tasks.

The agenda is as follows: Section 2 discusses Kernel-based covariance’s fundamentals that ground the Gaussian functional connectivity. Section 3 describes the experimental set-up, including the parameter tuning of time-frequency representations that were extracted from the EEG datasets evaluated. Section 4 presents the extraction of the compared connectivity measures and their influencing parameters on accuracy estimation, addressing subject clusters’ interpretation while using their functional connectivity patterns. Finally, Section 5 concludes the paper.

## 2. Methods

### 2.1. Kernel-Based Covariance Function

Let *x* be a wide-sense stationary stochastic process with real-valued auto-correlation function, $R_{x}\left(\tau \right)$, which is defined as below [33]:

(1) $$R_{x}\left(\tau \right)=\int_{R}\exp\left(j2\pi \tau f\right)dP_{x}\left(f\right),$$

where $P_{x}\left(f\right)\;\in \;R\left[0,1\right]$ is a monotonic spectral distribution function absolutely continuous and differentiable over frequency f∈R.

The univariable relationship in Equation (1) that operates over positive-definite functions can be expanded to a pairwise correlation between random vectors $x,x^{\prime }\;\in \;R^{T}$ through a generalized, kernel-based covariance, if and only if the following assumption holds between both spectral representations [34]:

(2) $$\kappa \left(x,x^{\prime }\right)=\int_{R}\exp\left(j2\pi {\Delta x}^{⊤}f\right)S_{xx^{\prime }}\left(f\right)df,$$

where $\Delta x=x-x{}^{\prime }$ is the vector delay that is defined over an infinitely long interval *T* (i.e., $\Delta x\;\in \;R^{T}$), f⊆Ω is the frequency domain that contains the bandwidth set of analysis Ω, and $S_{xx^{\prime }}\left(f\right)\;\in \;C$ is the cross-spectral density that preserves the following equality: $S_{xx^{{}^{\prime }}}\left(f\right)=dP_{xx^{{}^{\prime }}}\left(f\right)/df$, with $P_{xx^{\prime }}\left(f\right)\;\in \;R\left[0,1\right]$ being the cross-spectral distribution that is related to the mapping kernel, $\kappa \;:\;R^{T}\times R^{T}\to R$.

As regards $\kappa \left(x,x^{\prime }\right)={\langle \phi \left(x\right),\phi \left(x^{\prime }\right)\rangle }_{H}$, it is a positive-definite stationary kernel inducing the nonlinear feature mapping ϕ(·) to a Reproducing Kernel Hilbert Space, H. Notation 〈·,·〉 represents the dot product.

Therefore, we compute the cross-spectral distribution $P_{xx^{\prime }}\left(f\right)$ within a bandwidth Ω, as below:

(3) $$\begin{array}{cc} P_{xx^{\prime }}\left(f\right) & =2\int_{f\;\in \;\Omega }S_{xx^{\prime }}\left(f\right)df\\ \; & =2\int_{f\;\in \;\Omega }F\left\{\kappa (x,x^{\prime })\right\}df. \end{array}$$

Notation F{·} stands for the Fourier transform.

As a result, Equation (3) preserves the frequency-based interpretation of the kernel-based pairwise dependencies that were estimated between vectors of random functions. Furthermore, the imposed stationary kernel favors the extraction of nonlinear data dependencies [35].

### 2.2. Gaussian Functional Connectivity from Kernel-Based Spectral Distribution

Let $\left\{X_{rn}^{t}\;\in \;R^{C\times T},y_{r}\;\in \;R\left[0,1\right]:n\;\in \;\Omega ,t\;\in \;\Delta :r\;\in \;R\right\}$ be a EEG data that hold R∈N trials and C∈N channels within the frequency bandwidth set |Ω| and time window set |Δ|, lasting T∈N instants, and $y_{r}$ is the class-probability label set assumed to be binary without a loss of generality, and assigned to *r*-th trial. Notation |·| stands for set cardinality.

Provided the EEG data described above, we propose the Gaussian function to compute the pairwise kernel-based dependencies between electrodes $c,c^{\prime }\;\in \;C,c\neq c^{\prime }$ in Equation (2), termed Gaussian Functional Connectivity (GFC), defined as follows:

(4) $$\begin{array}{c} \kappa_{G}\left(x_{rn}^{ct}-x_{rn}^{c^{\prime }t};\sigma \right)=\exp\left(-∥x_{rn}^{ct}-x_{rn}^{c^{\prime }t}{∥}_{2}^{2}/2\sigma^{2}\right), \end{array}$$

where the vector $x_{rn}^{ct}\;\in \;R^{T}$ (with $X_{rn}^{t}=\;{\left[x_{rn}^{ct}\right]}^{⊤}$) denotes each filtered channel *c* of trial *r* within bandwidth *n*, at time window *t*, and $\sigma \;\in \;R^{+}$ is the length scale hyperparameter. Notation ${∥\cdot ∥}_{q}$ stands for $\ell_{q}$-norm. The Gaussian kernel is preferred in pattern classification because of its universal approximating ability and mathematical tractability [36].

With the aim to implement the single-trial extraction, we compute the pairwise functional connectivity matrix ${\hat{P}}_{n}\;\in \;R^{C\times C}$ as a kernel-based cross-spectral distribution estimator of Equation (3). Hence, from the spatio-temporal-frequency patterns, the functional connectivity matrix has elements that are extracted from *r*-th trial, as below:

(5) $${\hat{P}}_{cc^{\prime }}\left(r;u^{cc^{\prime }},\kappa_{G}\right)=\sum_{r\;\in \;\Omega }\sum_{t\;\in \;\Delta }u_{nt}^{cc^{\prime }}\kappa_{G}\left(x_{rn}^{ct}-x_{rn}^{c^{\prime }t};\sigma \right),$$

where $x_{rn}^{ct},x_{rn}^{c^{\prime }t}\;\in \;X_{rn}^{ct}$, ${\tilde{P}}_{cc^{\prime }}\left(r;u^{cc^{\prime }},\kappa_{G}\right)\;\in \;{\hat{P}}_{r}$ and $u_{nt}^{cc^{\prime }}\;\in \;u^{cc^{\prime }}$, with $u^{cc^{\prime }}\;\in \;R^{\left|\Omega |+|\Delta \right|}$, holds the relevance weight value that is estimated at t,n-th time-frequency split, coding the pairwise undirected dependency between channels.

Using the single-trial kernel-based spectral distribution representation in Equation (5), we propose extracting the sparse functional connectivity, aiming to finding discriminative and interpretable brain activity patterns. Particularly, the sparse-based $\ell_{2}$-norm matching is carried out after a vector concatenation of the relevance weights in $v=[u^{cc^{\prime }}]$, as follows:

(6) $$v^{*}=\arg\min_{v}E\left\{∥\sum_{c<c^{\prime }}{\tilde{P}}_{c,c^{\prime }}\left(r;u^{cc^{\prime }},\kappa_{G}\left(\cdot ,\sigma \right)\right)-y_{r}{∥}_{2}^{2}:\forall r,c,c^{\prime }\right\}+\alpha_{1}{∥v∥}_{1}+\alpha_{2}{∥v∥}_{2},$$

where $\alpha_{1},\alpha_{2}\;\in \;R^{+}$ are the regularization hyperparameters.

Consequently, the optimization framework in Equation (6) allows for computing the spectral combination weights from the extracted filter-bank representations that match the MI class-probability set, resulting in a sparse, relevant kernel-based functional connectivity between couples of EEG channels.

## 3. Experimental Set-Up

The evaluation of the considered functional connectivity measures for enhanced feature extraction is performed according to the following stages:

(i) Preprocessing and trial-based extraction of *t-f* representations. For extracting the subject EEG dynamics over time accurately, the sliding window length of feature extraction is fixed to the next values: τ=[0.5,1.0,1.5,2.0] s, having an overlap of 75%;

(ii) The estimation of the single-trial functional connectivity from the extracted *t-f* features. For comparison, we contrast the proposed GFC with two commonly used single-trial FC measures that can be estimated from a couple of channels $\left\{c,c^{\prime }\right\}$, (with $c\neq c^{\prime },\forall c,c^{\prime }\in C$), respectively, as follows [30]:

(7a) $$\begin{array}{cc} \rho (x_{n}^{ct},x_{n}^{c^{\prime }t}) & =\langle x_{n}^{ct},x_{n}^{c^{\prime }t}\rangle \end{array}$$

(7b) $$\begin{array}{cc} \Delta \phi (x_{n}^{ct},x_{n}^{c^{\prime }t}) & =\left|\exp(j\left(\phi_{n}^{ct}-\phi_{n}^{c^{\prime }t}\right))\right| \end{array}$$

where $x_{n}^{ct}$ and $x_{n}^{c^{\prime }t}$ is the real-valued EEG data from the corresponding electrodes (within temporal window *t* and frequency band *n*) with instantaneous phases $\phi_{n}^{ct}$ and $\phi_{n}^{c^{\prime }t}$, respectively. The pairwise relationships in Equations (7a) and (7b) are referred as Cross-Correlation Coefficient (CCF) and Phase Lag Value (PLV), respectively.

For feeding the classifier procedure, we evaluate two feature representation approaches that are extracted from each tested FC measure: Firstly, the feature representation is the CSP’s pattern vector (linear projection of the sample covariance), computed as detailed in [37]; in particular, the well-known CSP’s eigendecomposition is applied by replacing the covariance matrix with the corresponding FC measure. Secondly, the concatenated (vectorized) triangular representation of the symmetrical FC matrices is extracted, as detailed in [38]. Both of the approaches comprise a filter-bank-based concatenation strategy through a fixed sliding window size and overlap values; that is, the temporal dependencies between windows are not directly modeled.

(iii) Sparse feature selection and classifier performance computation. Here, for implementation purposes, the optimizing problem is solved through the well-known Elastic-net algorithm to deal appropriately with redundant information. It is worth noting that we prefer Elastic-net over the Lasso-based regularization, because, when the number of features is greater than the number of training samples, Lasso behaves erratically [39].

For comparative purposes, we also perform feature extraction using the baseline CSP-based spatial filtering that is widely used for filtering measures of synchronization [40,41]. To this end, we perform the CSP feature extraction, adjusting the sliding time window length at each evaluated value of τ and fixing the variance of the surrogate space to the first three eigenvectors of the spatial filtering matrix, as suggested in [42]. It is worth mentioning that we evaluate the FC measures in Equations (7a) and (7b), as well the CSP-based feature extraction scheme, through the sparse model presented in Equation (6) using a vector concatenation approach through temporal windows and frequency bands.

(iv) Clustering of subject inefficiency and interpretation analysis. For enhancing the interpretive analysis, we cluster the differences in neural responses that depend on the users’ motor skills. The intra/inter-subject variability affects the FC estimators’ robustness properties, becoming stronger for the worst-performing individuals.

Regarding the performance measure, the classifier accuracy $a_{c}\;\in \;\left[0,1\right]$ is computed, as follows:

$$a_{c}=\;\left(T_{P}\;\;+\;\;T_{N}\right)/\left(T_{P}\;\;+\;\;T_{N}\;\;+\;\;F_{P}\;\;+\;\;F_{N}\right),$$

where $T_{P}$, $T_{N}$, $F_{P}$, and $F_{N}$ are true-positives, true-negatives, false-positives, and false-negatives, respectively. The training and testing sets are randomly partitioned by a stratified 10-fold cross-validation strategy.

### 3.1. EEG Data Description

In order to appraise the properties of the single-trial Gaussian functional connectivity measure, we test the following databases of motor-related tasks:

**BCI2a Motor Imagery—DBI MI:** We test the BCI competition IV dataset IIa available at (http://www.bbci.de/competition/iv/index.html (accessed on 15 March 2021)). This competition contains EEG data from nine subjects (M=9) that were instructed to perform four MI tasks: left hand, right hand, feet, and tongue. Data were gathered in two days within six runs, yielding three runs per day. One run contains 12 trials per task, which were recorded by C=22 channels, for a total of R=144 trials of each label per subject where each channel was sampled at 250 Hz. The beginning of each trial, lasting T=7 s, was indicated by a short acoustic warning, and a cross on a black screen for 2 s, then an arrow pointing either left, right, down, or up appear to indicate subjects to perform the desired MI task. Besides, the subjects were requested to accomplish the MI task until the cross disappeared, six seconds later. Afterward, a black screen indicates a short break until the beep and the cross appeared again, and a new trial starts. In this study, we consider a bi-class (left and right hand) classification task.

**Gamma Motor Execution—DBII ME:** We explore the data that are publicly available at (https://gin.g-node.org/robintibor/high-gamma-dataset (accessed on 15 March 2021)). This collection is a 128-channel dataset sampled at 500 Hz (of which we later only use 44 sensors covering the motor cortex), obtained from 14 healthy subjects (6 female, 2 left-handed, age 27.2±3.6) with four-second trials of executed movements divided into 13 runs per subject and R=40 trials of each label per subject. The four classes were movements of either the left hand, the right hand, both feet, and rest (no movement, but the same type of visual cue as the other classes). Visual cues were presented while using a monitor outside the cabin, which was visible through the shielded window. A fixation point was attached at the center of the screen. The subjects were instructed to relax, fixate the fixation mark, and keep as still as possible during the motor execution task. The tasks were as follows: Depending on the direction of a gray arrow that was shown on the black background, the subjects had to clench their toes (downward arrow), repetitively, perform sequential finger-tapping of their left (leftward arrow) or right (rightward arrow) hand, or relax (upward arrow). The movements were selected to require little proximal muscular activity, while still being complex enough to keep subjects involved. Within the 4 s trials, the subjects performed the repetitive movements at their own pace, which had to be maintained as long as the arrow was showing. Per run, 80 arrows were displayed for 4 s each, with 3 to 4 s of continuous random inter-trial interval [43].

**Giga Motor Imagery—DBIII MI:** This collection publicly available at (http://gigadb.org/dataset/100295 (accessed on 15 March 2021)) holds EEG data that were obtained from fifty-two subjects (although only M=52 are available for evaluation) using a 10–10 placement electrode system with C=64 channels. Each channel x(c) lasted T=7 s, and it was sampled at $F_{s}=512$ Hz. At the beginning of the test, a fixation cross was displayed on a black screen during 2 s. Subsequently, being linked to either MI label [0,1], a cue instruction appeared randomly on the screen within 3 s. The cue asked each subject to imagine moving his fingers, starting to form the index finger and reaching the little finger, and touching each to his thumb. A blank screen was then displayed at the beginning of a break period, which ran randomly between 4.1 and 4.8 s. This procedure was repeated over 20 times to complete a single run and stopped at the end to complete a written cognitive quiz. Every subject performed between *R* = 100–120 trials of either labeled task, being acquired in five or six runs. In addition, a single-trial resting-state recording, lasting 60 s, was collected from each subject.

Of note, for evaluating the proposed connectivity measure, we test the baseline DBI MI data that are frequently used to appraise MI processing algorithms [44]. However, a reliable group analysis can barely be performed, since this collection holds few subjects. Thus, we also evaluate the frequently reported DBIII MI data with more subjects. Alongside the MI data, we present the experimental results on a similar motor execution paradigm, employing the DBII ME data as one of the most cited collections of ME [45].

### 3.2. Preprocessing and t-f Extraction

As a preprocessing stage, the raw EEG data of the three evaluated motor-related collections are band-pass filtered. In the particular case of MI data, we fix |Ω|=17 five-order overlapped Butterworth filters that contain bandpass frequencies between 4 Hz and 40 Hz, having a bandwidth of 4 Hz and overlapping rate of 2 Hz. In the case of ME, as suggested in [46], we fix |Ω|=4 with the following non-overlapped bandpass frequencies, f∈Ω: $f_{\alpha }$ = 8–12 Hz, $f_{\beta }$ = 12–30 Hz, $f_{\gamma }$ = 30–50 Hz, and $f_{\gamma }$ = 50–125 Hz. Next, the attained filter-banked featuresn that are extracted from all motor-related data are time-windowed onto |Δ| intervals with a 75% overlap of samples, and window size of τ s. Of note, the EEG data are not Laplacian-filtered, since there is no significant difference in the classification performance, as suggested in [47].

## 4. Results and Discussion

### 4.1. Influencing FC Parameters on Accuracy Estimation

**Figure 1:**
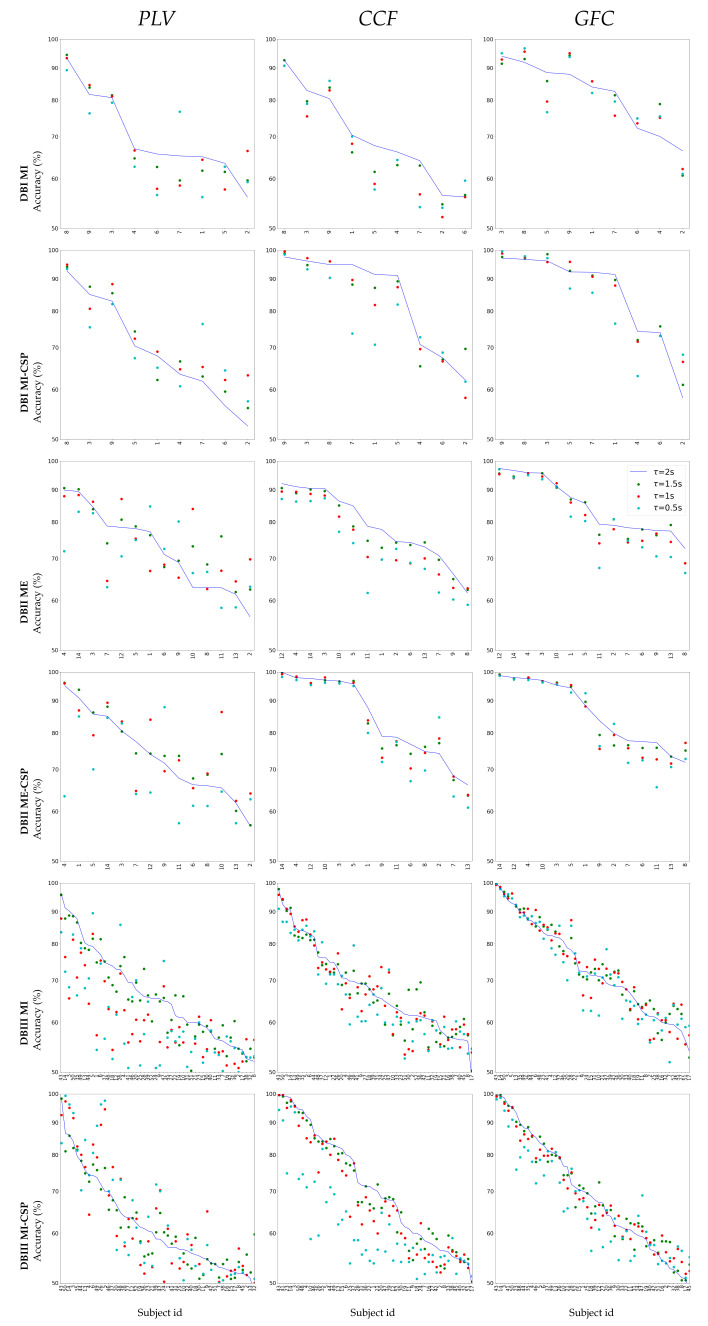
Subject accuracy at each window length τ performed by the evaluated FC measures: PLV, CCF, and GFC. Subjects are displayed on the horizontal axis in decreasing order of each FC accuracy at τ=2 s. Notation CSP stands for the accuracy estimated after the spatial CSP filtering.

*Impact of prior CSP filtering*: for interpretation purposes, Figure 1 presents the subjects’ accuracy in decreasing order by all evaluated EEG data collections. Each data set is tested in two configurations to feed the sparse feature selector: using feature concatenation (odd rows) and linearly mapping through CSP (even rows). The FC measures’ accuracy is estimated at each τ (*PLV*—left column, *CCF*—middle column, *GFC*—right column), adjusting the parameter σ to the median averaged over the input distances for kernel-based functional connectivity computation.

The top plots display the results that were performed by the databases with a similar amount of subjects: DMBI MI (first and second rows) and DB DBII ME (third and fourth rows). As seen, PLV yields the most scattered outcomes, with a significant portion accomplishing low accuracy. A similar situation holds for DBIII MI with a more visible subject variability: PLV discriminates the worst, followed by CFF, and GFC performs a bit better, still outperforming the other FC when compared and having the accuracy values less spread throughout the subject set. However, the accuracy estimates are significantly more dispersed across the individuals than in DBII ME, regardless of the FC measure. Several factors may account for the higher variability of MI data. The EEG montage of MI protocols is more complex (64 MI channels vs. 44 ME channels). Besides, the ME signals are reported as having a higher degree of regularity than MI practicing, at least in terms of motor-related discriminability [48]. Consequently, even if the DBIII MI collection doubles the number of trials compared with DBII ME, the subject variability of MI data remains high enough to limit the CSP effectiveness (see the bottom row). Moreover, numerous subjects achieve accuracy values that are below 60%, which is, under the BCI-inefficiency level reported [49].

**Figure 2:**
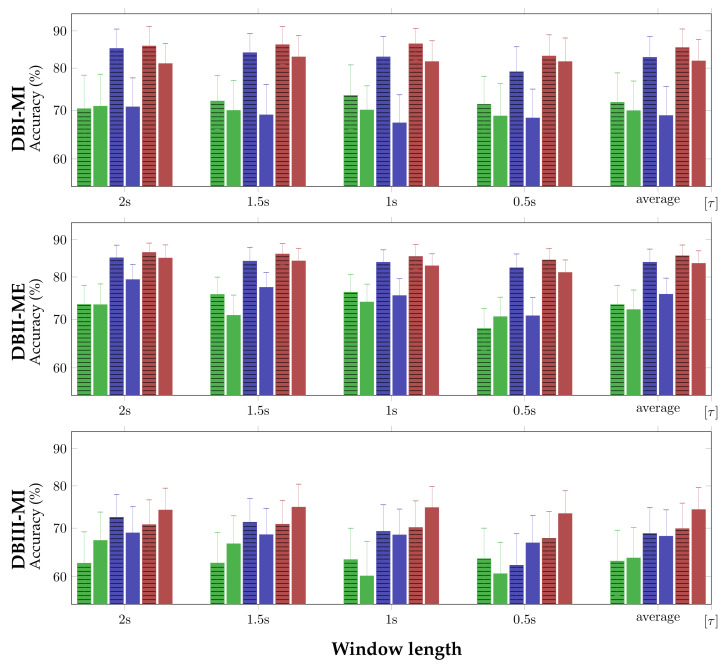
Accuracy performed at each τ by the evaluated measures of FC: PLV(▮), CCF(▮), and GFC (▮). The full-filled colored bars stand for concatenate representation, while the bars with horizontal lines present the results after CSP filtering.

*Influence of sliding window*: Figure 2 summarizes the sliding window’s impact on the classifier discrimination ability using each evaluated measure of FC. In the case of DBI MI, the first row shows that PLV and CCF both obtain very close performance if the CSP filtering is not involved at all tested window values. Otherwise, the latter measure notably improves in performance. Nevertheless, the proposed GFC outperforms, and this behavior remains valid in the second row that presents the accuracy of DBII ME with more subject variability, as shown by the average bars. However, some fluctuations in the PLV and CCF performance are more evident after applying CSP, depending on the window. Instead, the GFC measure presents performance nearly invariant across the range of considered sliding window lengths, being slightly enhanced by the CSP filtering algorithm.

On the other hand, the bottom row presents the classifier performance that is obtained by MI activity (DBIII MI) with the most challenging subject variability, showing that the accuracy of all FC measures drops, particularly in the case of PLV. As seen, the PLV and CCF accuracy values are more sensitive to the sliding window influence, while the proposed GFC assessments remain constant over the range of τ. One more aspect to highlight is that the CSP-based filtering effectiveness becomes more dependent on the specific sliding window selected. Furthermore, under increased subject variability, this spatial filtering method’s application noticeably decreases the GFC performance.

### 4.2. Estimated Classifier Accuracy of Individuals

**Figure 3:**
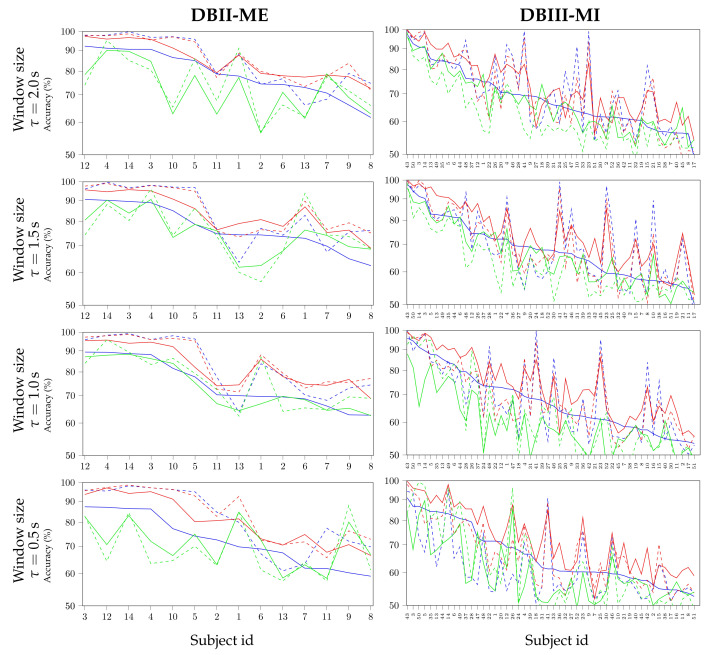
Individual classifier performance at different window lengths using FC: PLV (▬), CFF (▬), and GFC (▬). The accuracy assessments performed without CSP are outlined with a continuous line, after CSP filtering—with a dashed line. The subjects are displayed on the horizontal axis in decreasing order of their CCF-based accuracy.

The following consideration is linked to the quality of individual FC assessments because of their diverse variability. For this purpose, Figure 3 illustrates the individual classifier performance at each window length, for which the subjects are displayed on the horizontal axis in decreasing order of the CCF accuracy achieved at τ=2 s (the continuous line outlined in blue color). The rationale for choosing this specific FC estimation case as the baseline is that the cross-correlation coefficient that is presented in Equation (7a) can be directly associated with the conventional Pearson estimate with the simplest interpretation regarding pairwise relationships [50].

At first sight, besides being distinctive from the CCF and GCF, the variability of accuracy estimates that are achieved by PLV (green line) becomes visible across the subject set of DBII ME: the shorter the window length, the more variable the subject estimates. Rather, the CFF and GFC measures produce accuracy values following a similar order of subjects, for which the enhancing effect of CSP-based filtering can be observed. In the case of DBIII MI, the variability in classifier performance that is provided by the FC metrics grows noticeably, regardless of applying the CSP filtering. Thus, the order of subjects provided by each FC measure is particular and dissimilar to each other. Nevertheless, when compared with the baseline CCF-based accuracy (blue line), the proposed GCF measure without CSP allows for enhancing almost every single subject’s discrimination ability, regardless of the window length applied. Moreover, the kernel-based FC measure ensures that several subjects exceed the BCI-inefficiency level, as previously assessed using CCF. It is noteworthy that using the CSP algorithm appreciably reduces the GFC effectiveness for dealing with the subject variability of DBIII MI.

### 4.3. Interpretation of Subject Clusters Using Functional Connectivity Patterns

The main goal can be framed as the grouping of subjects having similar spatial networks involved in the motor-related tasks under consideration, aiming to properly interpret the evaluated functional connectivity measures. To this end, we cluster the extracted FC sets into partitions, each one with resembling subjects in terms of FC dynamics, being coded in the relevance vector v of Equation (6), and the motor-related accuracy. However, because of the poor clustering performance in high dimensional spaces, we accomplish a previous dimensional reduction stage on the normalized subject’s relevance vector set (fixing a 2D low-dimensionality) through t-Distributed Stochastic Neighbor Embedding (t-SNE), which preserves the spatial relationships in the higher space (nearest-neighbors) [51]. Subsequently, we concatenate the t-SNE low-dimensional representation with the corresponding individual accuracy to perform a *k*-means-based clustering. At each window length, the latter approach is carried out, as proposed in [52].

**Figure 4:**
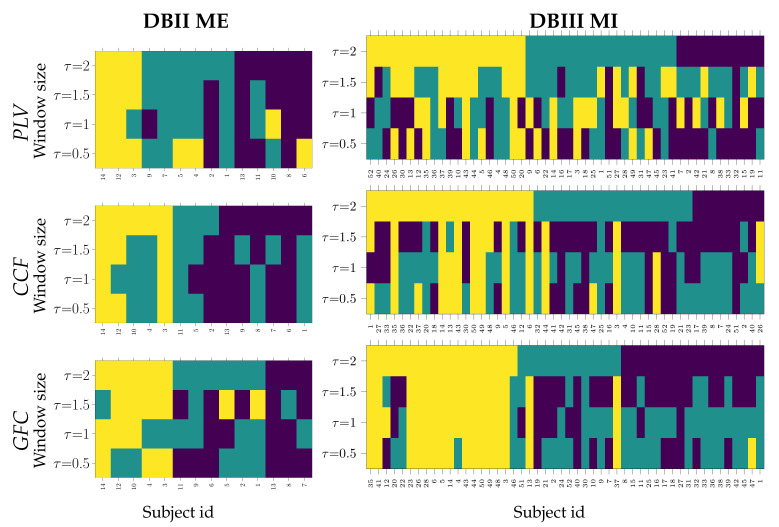
Clustering variability of individuals that belong to Group I (cells in yellow), Group II (Turquoise), and Group III (purple), depending on the extracting window length τ.

One concern is how a subject may switch between the assigned clusters when accounting for the influence of extracted FC measures at each window length. To clarify this aspect, Figure 4 displays the clustering results achieved by each functional connectivity measure using a matrix that illustrates the cells colored according to the individual group assessed, ranking the assessed groups in decreasing order of accuracy averaged over the corresponding subset. The three groups are colored, as follows: Group I, giving the best accuracy I (in yellow), Group II with regular accuracy (purple), and Group III, performing the worst accuracy (Turquoise).

As seen for DBII ME, the intragroup similarity remains comparable in groups I and III over the values of τ, while Group II has noticeable volatile behavior. This result may be explained because of the low number of database subjects (only 14), making any exchange seriously affect the intergroup distribution. In DBIII MI, the high subject variability that is reflected by the functional connectivity extracted yields high changeability between groups, at least for the PLV and CCF measures. Rather, the proposed GFC measure is less affected, as seen at window values of 2 and 1.5 s. Further, groups II and III become scrambled, meaning that the subject variability extracted at the shorter window lengths remains hard to overcome.

The last aspect of the study is the interpretability of motor-related tasks as reliable sources of neural responses in the assessed clusters of dynamic behavior (the groups are denoted as I, II, and III).

**Figure 5:**
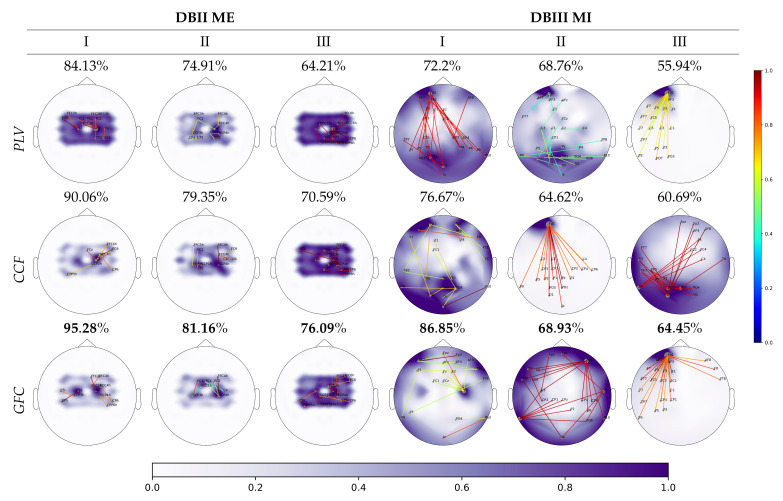
Spatial relevance mapped to topoplots estimated for each group of individuals from the FC measures (regarding the relevance weights v and the classification accuracy). The normalized relevance weights values (between 0 to 1) are computed from Equation (6). The background stands for the accumulated spatial relevance mapped to the EEG channel positions. The colored links represent the normalized FC relevance weights that hold a value higher than the 99 percentile of vector v. Above each plot, the average accuracy is shown over the corresponding group.

Figure 5 presents the topographic channel plots (topoplots) that contain the relevant activated scalp areas that are inferred from the extracted FC measures, principally contributing to the discriminability between labeled motor-related tasks. We also include the most relevant pairwise functional connectivity links between electrodes that are estimated to fulfill the 99 percentile of the normalized relevance weights values (between 0 to 1) computed from Equation (6). The background stands for the accumulated relevance that is mapped to the channel positions. In DBII ME, only the SMR area is depicted according to the electrode set configuration above-described.

PLV performs in DBII ME the first three plots in the top row. The topoplots reflect high evoked response amplitudes that are densely spread over the SMR area, which means that the PLV variability hampers the sparse feature selection framework to obtain a reduced set of extracted FC features. Still, a few links surpass the 99 percentile, which enables some information regarding the relevant electrodes. Contrary to this, the three topoplots that are delivered by CCF (middle row) and GFC (bottom row) show low amplitudes of neural activity in Group I, slightly increased activity of Group II, and increased amplitudes of the worst-performing group of individuals (III). However, the latter FC measure clearly shows two foci that are symmetrically located over each hemisphere, as expected in motor-related tasks, at least for GI and, to a less extent, for GII. Besides, the amount of relevant links estimated is low. Consequently, the sparse-based $\ell_{2}$-norm framework enables the effective dimensionality reduction of the extracted GFC features. Moreover, the accuracy using GFC assessed by every group of individuals outperforms the other compared functional connectivity metrics: PLV and CCF.

In DBIII ME, the full electrode montage is employed and, thus, the estimated neural responses are all spread over the scalp, as seen in the three last topoplots of every row. Likewise, the number of relevant links increases. Nonetheless, the compared FC measures’ performance has some similarities with DBII MI: PLV presents high background activity, CCF has amplitudes with fewer amplitudes, and GCF achieves a very focalized activity over the SMR hemispheres, which play a critical role in MI tasks. Once again, the proposed GFC measure outperforms the values of accuracy attained by other compared FC metrics. However, G III produces highly increased response amplitudes abnormally confined over the frontal zone (outside the SMR zone) and numerous links going to different electrodes. This issue may be explained because of acquisition artifacts, which may introduce noise and distortions, severely damaging the EEG data quality.

## 5. Concluding Remarks

We introduce a single-trial kernel-based functional connectivity measure to deal with inter/intra-subject variability in motor-related tasks. To this end, from the spatio-temporal-frequency patterns, we extract the functional connectivity between EEG channels through a novel kernel-based cross-spectral distribution estimator. Namely, the Gaussian kernel is used to compute the pairwise kernel-based channel dependencies because of its universal approximating ability. Further, we optimize the spectral combination weights within a sparse-based $\ell_{2}$-norm feature selection framework matching the motor-related labels to the extracted GFC features. The validation results that are accomplished in three EEG databases (two with MI and one with ME) show that the proposed single-trial connectivity measure allows for reaching very competitive classifier performance with less affected values by feature extraction parameters like the tuning of sliding time window length. The introduced connectivity measure does not demand a prior linear spatial filtering as a preprocessing procedure and holds spatial and connectivity interpretation. Additionally, the interpretation of subject clusters using our functional connectivity benefits from understanding the inter/intra-subject variability in motor-related tasks. From the obtained results, the following aspects are to be highlighted:

*Selecting the sliding time window.* The window length selected for extracting the EEG dynamics over time is a pivotal parameter. From the results obtained, both contrasted single-trial FC measures (Cross-Correlation Coefficient and Phase Lag Value) show several fluctuations over the evaluated values of τ that diminish the performed accuracy, becoming worse in databases with increased subject variability, as is the case of DBIII MI. On the other hand, the proposed Gaussian Functional Connectivity measure enables accuracy estimates that are less affected by the sliding time window and, thus, GFC allows for extracting the subject’s dynamics more accurately within wider ranges of τ.

*Prior spatial filtering versus to feed the concatenation of FC feature sets.* Because of the poor signal-to-noise ratio of scalp EEG measurements, the baseline CSP-based spatial filtering is very frequently accomplished. Meanwhile, the evaluating results indicate that the CSP effectiveness degrades noticeably as the inter/intrasubject variability increases. Thus, a big partition of subjects in DBIII MI turns out to be below the BCI-inefficiency level. These findings are according to the reported CSP variability from trial-to-trial [53], and the subject-dependent choice of its extraction window [37].

One more restriction is that CSP is more oriented to power-based features [41], so that its validity of the combination with PLV is questionable, as the achieved results definitely indicate. The combination of CSP with CCF and GFC allows for enhancing the performed accuracy, but in DBI MI and DBII ME with a relatively moderate number of subjects. The testing of DBIII MI with the largest considered intra/inter-subject variability tends to nullify CSP filtering before CCF feature extraction. Moreover, the proposed GFC measures’ performance improves if avoiding using the spatial filtering algorithm, which means that FC feature sets’ straightforward concatenation is enough to feed the sparse-based $\ell_{2}$-norm feature selection framework. Table 1 displays the evaluated MI data’s accuracy (i.e., DBI MI, and DBIII MI) using functional connectivity measures that have been reported recently, noting that the proposed GFC approach provides very competitive classifier performance values.

*Interpretability of clustered functional connectivity patterns.* We validate the suitability for interpreting the extracted feature sets concerning the spatial networks that are assessed by the introduced sparse feature selection framework. To achieve this particular aim, we perform the clustering of the extracted FC measures together with the corresponding accuracy outcomes into three partitions of individuals with similar FC dynamics that are evoked by the motor-related tasks. This approach of clustering is increasingly accepted for appraising the BCI-inefficiency. In this regard, the benefit of interpreting PLV sets seems to be limited by its vulnerability to the subject variability, resulting in blurry topographic channel representations. Besides, PLV provides the worst classifier performance. Rather, the application of the CCF method results in topoplots with increased activity over the SMR electrodes. However, the proposed GFC measure clearly represents two foci over SMR electrodes that are symmetrically located over each hemisphere as expected in motor-related tasks. At the same time, the accuracy using GFC assessed by every group of individuals outperforms the other compared functional connectivity metrics. Therefore, we hypothesize that the application of single-trial kernel-based functional connectivity for evaluating motor skills is more promising.

The authors plan to enhance the Gaussian functional connectivity that was developed for feature extraction as future work, allowing a better understanding of their impact and interaction on BCI-related tasks. To identify potential non-learners, the efforts can be directed toward a twofold aim: to enhance the feature extraction by profiting from more elaborate methods for measuring multivariate similarity, like centered kernel alignment [58,59], and to explore the robust estimation approaches based on information metrics (like correntropy) for dealing better with the variability [55,60,61]. Besides, modeling the temporal-dependencies within each trail to compute the FC is an exciting research line.

## 6. Author Resume

- **Daniel Guillermo García-Murillo** received his undergraduate degree in electronic engineering (2017) and his M.Sc. degree in engineering industrial automation (2019) from the Universidad Nacional de Colombia. Currently, he is a PhD student at the same university. His research interests include machine learning, image processing, and bioengineering.
- **Andres Alvarez-Meza** received his undergraduate degree in electronic engineering (2009), his M.Sc. degree in engineering industrial automation (2011), and his Ph.D. in engineering—automatics (2015) from the Universidad Nacional de Colombia. Currently, he is a Professor in the Department of Electrical, Electronic, and Computation Engineering at the Universidad Nacional de Colombia – Manizales. His research interests include machine learning and signal processing.
- **German Castellanos-Dominguez** received his undergraduate degree in radiotechnical systems and his Ph.D. in processing devices and systems from the Moscow Technical University of communications and Informatics, in 1985 and 1990 respectively. Currently, he is a Professor in the Department of Electrical, Electronic, and Computation Engineering at the Universidad Nacional de Colombia, Manizales. In addition, he is Chairman of the GCPDS at the same university. His teaching and research interests include information and signal theory, digital signal processing, and bioengineering.

## Figures and Tables

**Table 1:** Classifier accuracy comparison of approaches using functional connectivity features recently reported against the Gaussian FC performance in discriminating MI tasks. Notation TSGSP is temporally constrained sparse group spatial pattern, STR is space-time recurrence, and OPTICAL is Optimized CSP with long short term memory (LSTM). The best value performed for each database is marked in bold.

Data	Time Window	Filter Band	Interpretation	Feature Extraction	Accuracy (%)
DBI-MI	✓	✓	✓	TSGSP [54]	**82.50** ± **12.2**
	-	-	✓	STR connectivity [30]	69.56 ± 15.02
	✓	-	✓	Renyi’s α-entropy [55]	72.40 ± 6.50
	✓	✓	✓	*Proposed GFC*	81.92 ± 9.44
DBIII-MI	-	✓	✓	CSP [56]	67.60 ± 13.17
	✓	✓	-	OPTICAL [57]	68.19 ± 9.36
	-	-	✓	STR connectivity [30]	62.00 ± 13.00
	✓	✓	✓	*Proposed GFC*	**74.12** ± **12.13**

## Data Availability

The databases used in this study are public and can be found at the following links: DBI http://www.bbci.de/competition/iv/index.html (accessed on 15 March 2021), DBII https://gin.g-node.org/robintibor/high-gamma-dataset (accessed on 15 March 2021), and DBIII http://gigadb.org/dataset/100295 (accessed on 15 March 2021),
